# What's in a message? Delivering sexual health promotion to young people in Australia via text messaging

**DOI:** 10.1186/1471-2458-10-792

**Published:** 2010-12-29

**Authors:** Judy Gold, Megan SC Lim, Margaret E Hellard, Jane S Hocking, Louise Keogh

**Affiliations:** 1 Centre for Population Health, Burnet Institute, Melbourne, Victoria, Australia; 2 Department of Epidemiology and Preventive Medicine, Monash University, Melbourne, Victoria, Australia; 3 The Nossal Institute for Global Health, The University of Melbourne, Melbourne, Victoria, Australia; 4 Centre for Women's Health, Gender and Society, The University of Melbourne, Melbourne, Victoria, Australia

## Abstract

**Background:**

Advances in communication technologies have dramatically changed how individuals access information and communicate. Recent studies have found that mobile phone text messages (SMS) can be used successfully for short-term behaviour change. However there is no published information examining the acceptability, utility and efficacy of different characteristics of health promotion SMS. This paper presents the results of evaluation focus groups among participants who received twelve sexual health related SMS as part of a study examining the impact of text messaging for sexual health promotion to on young people in Victoria, Australia.

**Methods:**

Eight gender-segregated focus groups were held with 21 males and 22 females in August 2008. Transcripts of audio recordings were analysed using thematic analysis. Data were coded under one or more themes.

**Results:**

Text messages were viewed as an acceptable and 'personal' means of health promotion, with participants particularly valuing the informal language. There was a preference for messages that were positive, relevant and short and for messages to cover a variety of topics. Participants were more likely to remember and share messages that were funny, rhymed and/or tied into particular annual events. The message broadcasting, generally fortnightly on Friday afternoons, was viewed as appropriate. Participants said the messages provided new information, a reminder of existing information and reduced apprehension about testing for sexually transmitted infections.

**Conclusions:**

Mobile phones, in particular SMS, offer health promoters an exciting opportunity to engage personally with a huge number of individuals for low cost. The key elements emerging from this evaluation, such as message style, language and broadcast schedule are directly relevant to future studies using SMS for health promotion, as well as for future health promotion interventions in other mediums that require short formats, such as social networking sites.

## Background

Over the past two decades communication has changed beyond imagination. Mobile phones, portable computing devices and the internet have all become widely accessible and provide entirely new avenues to access information, connect, and communicate regardless of geographic location. This rapid change in communications can be understood using Giddens's notion of the 'discontinuities of modernity'; contemporary society is characterised by the rapid pace and scope of change and changing nature of modern institutions [1]. Alongside these discontinuities, Giddens identifies reflexivity as a key feature of modern life. The proliferation of modes and speed of communication and the reflexivity of knowledge all have important implications for health promotion. Individuals continually examine and change their practices in light of incoming information from a variety of sources [1]. How best to present and deliver information in this rapidly changing environment is a key challenge for health promoters.

As the use of newer communication technologies continues to exponentially increase, health promotion will inevitably expand out from the 'old' media (TV, radio, billboards) and into the 'new' (mobile telephones, social networking sites). Text messages (SMS) are a highly promising method of health promotion for multiple reasons. They are widely available and accessible; in 2009 it was estimated that there were 3.6 billion global users of SMS, double the number of internet users [2]. Most mobile phone users have their phones turned on, and in reach, during waking hours. Messages can be sent to multiple recipients simultaneously and are delivered immediately. The delivery of messages to individuals can be tracked and is guaranteed, and the cost of sending messages is relatively low (generally less than USD 20 cents per message)[3]. Using SMS for health promotion is particularly appealing for reaching healthy individuals not regularly in contact with health services, and for behaviours that may be socially sensitive, as they offer a confidential, non-confrontational means of communication.

Recent reviews have concluded that SMS can be used successfully to promote behaviour change in the short term (one year or less) for several behaviours,[4,5] including smoking, diet and physical activity [4,5]. Behaviour change interventions using SMS have generally provided participants with information and reminders relevant to the behaviour of interest, Approaches to designing and delivering the text messages include relatively simple systems, where the same message is sent out to each participant,[6-9]as well as systems where broadcasts are individually tailored to participants characteristics and preferences [10-14], whether they reply to messages [15] or both [16]. A recent review identified the lack of knowledge about optimising and enhancing the use of SMS for health behaviour change interventions [5]. As text messages become more widely used for health promotion it is critical to understand what characteristics affect the acceptability, utility and efficacy of messages.

A randomised controlled trial we conducted in 2006-2007 found those receiving SMS and email messages about sexual health improved their knowledge, and that females receiving messages were more likely to seek an test for sexually transmitted infections (STIs) than those who did not receive any messages [8]. Subsequently, we scaled up this approach in the "SMS 2008 project" to determine the impact of using SMS on a population level on sexual health knowledge and behaviour using a pre and post evaluation design. The SMS 2008 project targeted young people aged 16 to 29 years recruited at a music festival in Melbourne, Australia; participants (n = 1,771) received fortnightly SMS relating to sexual health for four months [17]. The quantitative evaluation of the project found a significant improvement in sexual health knowledge and an increase in the proportion tested for STIs after receiving the messages [17]. Here we present the results of the focus group evaluation, designed to examine the characteristics of the messages that affected acceptability and efficacy of the messages in promoting behaviour change.

## Methods

### Recruitment

All participants in the SMS 2008 project were aged 16 to 29 years when enrolled at baseline in January 2008. The twelve messages that formed the SMS intervention were broadcast in February to July 2008 (Table 1). At the conclusion of the broadcasts participants were asked to complete an online follow up survey to evaluate the intervention.

**Table 1:** Text Messages Broadcast

	Broadcast Date	Message Text
1^	Friday, 1 February 2008	Big Day Out! Big Night In? Forgot to use your free condoms? Speak to your doctor about a chlamydia test. Love from the Burnet Institute

2	Thursday, 14 February 2008 *(Valentine's Day)*	Roses are red, daisies are white, use a condom if you get lucky tonight. Happy Valentines Day! Love the Burnet Institute

3	Friday, 29 February 2008 *(Leap year)*	Unlike February the 29th, having chlamydia is common. More than 50,000 Australians were diagnosed with chlamydia last year. Love the Burnet Institute.

4	Friday, 17 March 2008 (Easter long weekend*)	Protect your or your partners eggs this Easter with a condom. Chlamydia can cause infertility. Enjoy the long weekend! Burnet Institute

5	Tuesday, 1 April 2008 *(April Fools Day)*	Dont be fooled, chlamydia testing and treatment is easy. Its just a pee and a pill, see your doctor today. Love the Burnet Institute

6^~^	Thursday, 10 April 2008 (End of daylight savings*)	Change your clocks, change your smoke detector battery. Change your partner, get an STI test. Love the Burnet Institute

7^#^	Tuesday, 29 April 2008	I know ur hurting, feels like ur burning - but maybe not? Dont be the Biggest Loser! Most STIs have NO symptoms, only way to know is to get tested.

8	Friday, 9 May 2008 *(Mother's Day)*	Spare a thought for condoms this Sunday, they can help you have babies too (chlamydia causes infertility). Burnet Institute PS Dont forget to call your mum

9	Friday, 23 May 2008	Well before the Big Day Out, its time to clear chlamydia out. Pap smears and blood tests are not the go, you need to pee in order to know. Burnet Institute

10	Friday, 6 June 2008	Chlamydia: hard to spell, easy to catch. Use a condom! Burnet Institute

11	Friday, 20 June 2008	Why did the chicken cross the road? Coz it realised a pap smear or a blood test didnt test it for chlamydia. Urine samples are the best chlamydia test.

12	Friday, 4 July 2008	Get those dancing shoes on, it takes two to tango! Pill for pregnancy, condoms for chlamydia. Last SMS from us but stay tuned for survey and prizes next week ...

^ This message refers to the Big Day Out music festival where participants received free condoms and were recruited for the study

* These were broadcast in advance of the event itself

~ This message is a play on a well known Australian ad with the slogan "change your clocks, change your smoke alarm battery"

# This messages plays on the theme song of the television show "The Biggest Loser", popular at the time

Those who completed the online follow up survey (n = 676) were asked if they consented to be contacted for future evaluation of the project. From the 676, 369 (55%) consented to be contacted. Selection criteria for the focus groups was being aged 16 to 24 years, the age group for which the intervention was most relevant; we aimed to recruit an equal number of males and females. Among the 289 16-24 year olds who had consented to be contacted, we attempted to contact 162 (56%) and successfully contacted 141 (49%); the remaining individuals were not contacted as sufficient participants had been recruited for the eight focus groups planned.

### Data Collection

Focus groups were held in private meeting rooms at one urban and one regional site (Melbourne and Ballarat) and lasted no more than one hour. To encourage full participation, separate groups were held for males and females. Participants were provided with a participant information and consent form and were required to read and sign the consent form before the discussion commenced. Participants were provided with refreshments during the focus group, were reimbursed AUD $30 (USD $25) for their time and travelling expenses and sexual health information and condoms were distributed at the conclusion of the discussion. All discussions were audio recorded.

A focus group schedule was developed to determine what participants thought of the messages, and what, if any, impact the messages had on their sexual health knowledge and behaviour. Participants were asked what they thought of the messages in general, and of each message individually. Participants were prompted to comment on what they liked and disliked about the messages, and which messages they remembered receiving and why. They were also asked whether or not they thought the information contained within the message was important and if the messages had any impact on their sexual health knowledge and behaviour. Finally, participants were asked to comment on what could be improved for a similar project in the future, both in terms of the content and format of the messages themselves, and the broadcasting schedule. The same questions were asked at each focus group, although the order of the questions, and the specific wording of the questions, varied slightly from group to group.

Author JG facilitated all the focus groups, with another author (ML, JH or LK) also in attendance at each group.

### Data Management & Analysis

Audio recordings were transcribed verbatim, with names replaced with pseudonyms to protect confidentiality. Transcripts were imported into NVivo 8 for coding and analysis [18]. Transcripts were read and reviewed multiple times by author JG before analysis commenced. Thematic analysis was used, with data coded under one or more themes. The themes were pre-existing from the focus group schedule, with new themes identified and added during the analysis. Data was scrutinized for differences and similarities within themes [19].

An iterative analysis process was used, with author JG performing the coding, discussing the coding with author LK and then refining as necessary. Illustrative quotations for each theme were selected by authors JG and LK. Responses from male and female focus groups were generally similar; any differences found by gender are specifically noted. All names included in this manuscript are pseudonyms.

### Ethics

Approval for the evaluation focus groups was sought and granted from the Alfred Hospital Human Ethics Committee (located in Melbourne, Australia).

## Results

The main themes emerging from the focus groups are presented in Table 2.

**Table 2:** Key Themes

Message Style, Content & Delivery	Impact of the Messages
**Theme One: Style**	**Theme One: Information**
• Humour and rhyming	• New and Specific Information
• Variety	• Reminder
• Message length	**Theme Two: Behaviour Change**
• Event tie in	• No direct effect
• Sign off	• Perceptions of STI testing
**Theme Two: Language**	• Consider risk
• Informal	**Theme Three: Spreading the Word**
• Positive	• When and Why
• Indirect vs Direct	• Reactions
• Fear Factor	
• Use of statistics	
**Theme Three: Content**	
• Repetition	
• Relevance	
• Ease of understanding	
• Different	
**Theme Four: Broadcast Schedule**	
• Message Timing	
• Message Frequency	

### Participation

Among the 141 individuals successfully contacted, 108 (77%) were interested in participating in a focus group. There was no significant difference by gender or region of residence for expressing interest in participating (data not shown). Forty three individuals, 21 males and 22 females aged 16 between 24 years, attended one of the eight focus groups held in August 2008. The size of each focus group conducted ranged from four to seven participants. Six focus group discussions were held in central Melbourne, and two in Ballarat, a large regional centre.

### Message Style, Content & Delivery

When participants were asked what they through of the messages in general, a number of participants commented that they liked receiving the messages. Although not asked directly, several participants said they like receiving health promotion messages via SMS, as it felt more personal and informal than receiving messages in other forms.

It's different ... It's a new take on rather than sort of seeing sort of posters or billboards like everywhere, bombarding you ... because it's in a text, more personal. (Rick, 22 years)

And especially because it was being told to you over SMS it like made it ... made it more casual, it wasn't a doctor or a teacher. (Christina, 17 years)

The remaining comments related to the messages could be categorised into the following four broad themes: message style, language, content and broadcast schedule.

#### Theme One: Style

While discussing the messages overall, and individual messages specifically, a number of elements related to the style of the messages emerged in all focus groups. These included the use of humour and rhyming the messages, the variety and length of the messages, tie in with events and the 'sign off' used.

##### Humour and Rhyming

When responding to what they liked about the messages, participants in all focus groups commented how the humour and rhyming in the messages made them more likely to pay attention to the message and to remember the message and its contents.

It was sort of like, I'd open it and I'd be like, I really don't care about sexual education at the moment, but because it was funny, it just sort of stuck anyway and it's like, information gets through. (Pete, 17 years)

The ones that rhymed are really easy to remember (Tracy, 17 years)

##### Variety

Some participants noted they liked the variety in the messages sent, in particular the balance between information and humour.

.. That what was really good about the SMS thing. There were some that, like the 50,000 Australians every year, wow that's a lot of people you know, and you might not even know about it. And then there were other ones that were just really lighthearted, "hey you know, remember to use a condom" sort of thing. So it was a good balance. (Natalie, 18 years)

##### Message Length

Messages varied in length from 71 characters to 160 characters, the maximum allowed in the SMS format. There was no direct question about message length, but a number of comments emerged related to length when reviewing individual messages.

Many participants liked the shorter messages, finding them more straightforward and easier to remember.

[Re Message 10] *I, I reckon it's just good because it's just short, sharp. (Luke, 18 years)*

Opinions varied about the longer messages - some liked them and acknowledged the need for the extra length, whereas others found them too long which lessened their impact.

Tracy (17 years): Like short and to the point

*Christina (17 years): Its easy to remember*...

*Meg (16 years): Still, some of the long messages are like, very effective though. It's a mixture*...

##### Event Tie In

Participants in all eight focus groups commented that they liked that some of the messages tied into particular annual events, such as Valentine's Day and Easter, and tended to remember these messages the most.

...because you can associate it with something I think it's just more memorable. (Luke, 18 years)

##### Sign Off

When reviewing the individual messages, a number of participants commented they found the sign off of many of the messages "Love the Burnet Institute" made the messages more personal, and gave the message credibility.

I know it was good they all ended with "Love from the Burnet Institute" rather than just a random message because otherwise I would have wondered "Who the hell is this??!" (Natalie, 18 years)

#### Theme Two: Language

Along with style, language was another theme that emerged from all groups when reviewing the messages. Most prominent were comments related to the informality of the language use, the positive framing of the messages and the use of indirect versus direct language. Other elements of language that were commented on included the use of 'fear factor' and statistics.

##### Informal

Many participants commented they liked the informal language used in the message, which resonated with them.

Natalie (18 years): Its not like one of those things the teachers say "When you are having intercourse make sure..." its "If you get lucky..." [laughs]

Belinda (20 years): Its more like our language, than our teachers language in a way

However some participants thought the combination of informal language and serious content wasn't appropriate.

[Re Message Four]

Barry (20 years): I don't like the bit about the long weekend afterwards ... have a nice weekend. It's putting a dampener on things

*Mark (24 years): I think it's, it's too far. Like it's too laid back to the point of this isn't a serious issue*.

##### Positive

Another recurring sentiment that emerged while reviewing the messages was the positive angle of the messages, which was liked by a number of participants.

....it's not like, "use a condom or you're going to die!", it's like, "hey if you're lucky enough to get sex, why don't you use a condom?!" (Kate, 17 years)

##### Indirect vs. Direct

A third recurring emerging sentiment related to the use of indirect or direct language. Some participants felt the messages were quite indirect, and liked this.

Its not telling you to do something, its kind of like you want to do something. (Sarah, 20 years)

Other participants found the messages quite direct.

It was like every message accused you of having chlamydia [laughter] (Mick, 23 years)

However there was a sense that the directness could be interpreted as positive or negative - some liked it, and thought this approach would act as a good motivation, while others thought it could be seen as offensive.

[Re Message Six] *Change your partner ... it makes it sound like changing your partner is an everyday kind of thing ... It's amusing .....[but] some people would probably get offended by that. (Kenny, 18 years)*

##### Fear Factor

In one focus group there was a lot of discussion about mixing the informality of the language with an element of 'fear' to prompt action, and whether this was effective or not.

I think its really difficult, because one of the best ways to get through to people is through fear ... ....but, I mean saying "you could become infertile" is not going to make people want to go out and find out if they've got chlamydia. But at the same time, if you say "its not really that big a deal, you know, its easy to treat" well then they are going to go "well don't worry about it". Yeah, so its difficult either way (Natalie, 18 years)

Others though the use of fear wouldn't be effective.

And its kind of one of those things you know, all those ads that just say all those statistics and try to scare you and just like yeah, whatever, I can't be bothered (Rebecca, 18 years)

##### Use of Statistics

Only message three included a statistic. When reviewing this message, opinions varied greatly as to whether this was effective to include. Some found the statistic impressive and important to include.

[Re Message Three] *I reckon it's a good one ... it kind of does suggest that a lot of people do have it, so you might want to be a bit careful (Kate, 17 years)*

Others didn't like the use of the statistic, finding it boring, not memorable and not relevant personally.

That's like the Easter one, protect you and your partners eggs. It was like, it was hitting home, look after yourself rather then all that 50,000 Australians, I don't know them! [laughter] (Michalea, 22 years)

When asked how the messages could be improved, participants in three of the focus groups specifically nominated making the messages more personal, rather than the more generic statistics, and that this would have a bigger impact.

Maybe make them more personal to you, like if in the message if it says something like, I dunno, like it said think about 10 of your friends, one of you will get chlamydia, you're going to remember it. (Meg, 16 years)

#### Theme Three: Content

Comments related to message content emerged both out of direct questioning related to opinions of the overall content of the messages, as well as during review of the individual messages. The strongest theme to emerge related to content was repetition, specifically the focus of many of the messages on chlamydia. Other elements related to content include content relevance, ease of understanding, and the 'different' take of one particular message.

##### Repetition

A few male participants remarked they found the messages repetitive.

*Ben (18 years): Yeah with the repetitive thing it sort of, I mean, like, sort of got bit old, just the way there wasn't really anything new to it*.

Participants in all focus groups commented there was too much focus on chlamydia in the messages. This repetition made them pay less attention to the message content, and it would be more effective to include information about a wider range of topics in the future.

I think, look so many were just about chlamydia, I think if they had different information every time, quite clearly different, then I reckon it would have been more, like less annoying, because it was new (Jim, 17 years)

However others thought that the repeated focus on chlamydia was effective in building their knowledge about the infection.

I don't think necessarily it was too much [focus on chlamydia]. Like I feel like I know a fair bit about--now you know (Rob, 19 years)

##### Relevance

Some participants reported they found the messages very relevant to themselves and their peers.

Yeah, I think they, I think they were on the money ... geared towards the age that you're working towards. Like I think they were pretty accurate and resonated with everyone (Luke, 18 years)

Others remarked they didn't think the messages were relevant to everyone, and this may have affected how they reacted to the messages.

Oh, I was aware that there was a lot of sort of illnesses and that sort of thing which there aren't any symptoms for but um, I'm pretty confident that I wouldn't be affected at the moment so I've got nothing to worry about personally. And then, I suppose, in that case I haven't taken as much notice to these messages. (Paul, 20 years)

##### Ease of Understanding

When reviewing the individual messages, a number of participants commented they found some of the messages hard to understand, which reduced their effectiveness.

There were some that were okay but then ... there was a few that I found that were a bit round about. Um, I wasn't--they were a bit hard to follow. (Carol, 22 years)

##### Different

A few participants commented that they liked, and specifically remembered, the mother's day message that had a different take on condoms from the 'standard' health promotion messages.

[Re Message Eight] *Yeah and I like the way that one makes you think because it turns it around, saying condoms can help you have kids. Because normally it's just like, you wear a condom, you don't want to have kids kind of thing. (Kate, 17 years)*

#### Theme Four: Broadcast Schedule

Participants were asked to comment when and how often they recalled the messages coming, whether this was appropriate, and if and how this should be changed in the future. SMS were always sent in the afternoon, generally either on Friday's, or the day of a particular event (e.g. on Valentine's Day). Messages were broadcast approximately fortnightly (Table 1).

##### Message Timing

Many participants recalled that most messages came on Friday afternoons, and thought that this was an appropriate time.

*Mark (24 years): Friday afternoon seems to work because it's like, you know, easing into the weekend*.

*Paul (20 years): Yeah, you're in a good mood anyway*.

Some participants didn't particularly notice when the messages were arriving, and liked this.

If you had recorded dates it would have been boring. Because like it could be any time of day and I'd just be like, hey! (Bec, 18 years)

In terms of alternate times for message broadcasts, several participants suggested it would be beneficial to have messages sent 'after the fact' (on weekend mornings or Monday's). However others thought this wouldn't be an effective strategy.

##### Message Frequency

When prompted, the majority of participants recalled the messages as coming approximately monthly (not fortnightly), but felt the frequency was appropriate.

And they were nicely spread, it wasn't like, you know, all the time, like bombarding. That would have got annoying (Karen, 18 years)

A minority of male participants felt the messages came too frequently, and became too routine.

Yeah, but like toward--like, you know, it started being a routine thing. You're like, you know, they should be messaging me pretty soon about this. And then you do and you're like oh, yeah. So I think that could be why. It became a bit routine. (Josh, 23 years)

### Impact of the Messages

As well as gaining an understanding of what characteristics of the messages participants liked, the focus group discussions were also structured to examine if and how the messages had an effect on participants knowledge and behaviour as has been demonstrated by quantitative findings [17]. Given the group setting, we did not require participants to detail exactly how the messages impacted them (if at all), however a number of participants volunteered how they perceived the messages affected them.

The impact of the messages was broadly categorised into information and behaviour change, as well as sharing the messages with others.

#### Theme One: Information

Information emerged as a key impact of the messages, both the provision of new and specific information and a reminder of information already known to participants.

##### New and Specific Information

Participants in all focus groups commented they learnt new information from the messages, particularly about chlamydia causing infertility, and often not having any symptoms. Some remarked they liked how the SMS gave them specific information, particularly about STI transmission and methods of STI testing, that they didn't know previously.

Another one I remember was like ... to protect you from being able to have children in the future or something. That was actually new to me. (Pete, 17 years)

... the bits about the chlamydia testing and everything, I had no idea how it got tested or anything like that. (Tracy, 17 years)

Highlighting the value of the messages even further, several participants thought that they wouldn't have found out the information that was new to them in other ways.

Like it's not kind of information you really get um, through other sources really (Freddy, 20 years)

##### Reminder

Alongside new information, participants in all focus groups also reported that some messages acted as a reminder them of things they already knew.

Yeah.... I don't know how much of it was new information. It was like, jogging my memory sort of (Pete, 17 years)

Several participants commented they didn't mind being reminded of information they already knew.

It was like, some of it was stuff you already knew but it was like, cause they were funny, they weren't like up in your face kind of thing, it was just a reminder (Kate, 17 years)

One participant thought being reminded about information was really useful.

.. it's kind of like you need to be reminded ... Because there's knowing something and then being reminded about it as well. (Manisha, 22 years)

A number of participants commented the information they were reminded about was information they had originally learnt in school.

I think I did [know chlamydia causes infertility]. I think we did get taught something like that in school but like you don't really listen much in school, it all kind of blurs together (Christina, 17 years)

#### Theme Two: Behaviour Change

Alongside provision of information, the messages aimed primarily to change two key behaviours; 1) encourage uptake of STI testing and 2) reinforce consistent condom use. Participants generally viewed the messages as having no direct impact on their behaviour, but may have had indirect effects, such as reducing apprehension of STI testing and causing them to consider their risk of contracting an STI.

##### No Direct Effect

Many participants stated they didn't think the messages had a direct effect on their behaviour related to STI testing or condom use, especially if they thought they already were doing the things they should already.

Yeah, I was--I don't think I like I particularly thought I better put on a condom because of those messages. (Luke, 18 years)

Um ... I haven't really changed anything because pretty much everything that I learnt from the SMS I sort of already know before and taken whatever measures, things like that. (Natalie, 18 years)

However a number of participants thought the messages may have had indirect effects, particularly in keeping sexual health 'up in their mind'.

....it was regular, it kept me on my toes a bit, because if you don't have that.... I mean you don't see billboards of it so if you don't hear it from your mum, you just don't hear it. (Steph, 21 years)

When asked to reflect what had directly caused behaviour change in the past, participants in all groups described the impact of a crisis situation that had occurred, either to themselves or to a friend.

... I've had like little scares along the way which have made me go "I really need to use more condoms, this is ridiculous" (Belinda, 20 years)

... a friend of mine thought she was pregnant for like, for a while and that sort of like shocked her into being more careful. Whereas, you know, a cute SMS will never, can never really have the same impact (Chris, 20 years)

##### Perceptions of STI testing

Participants in almost all focus groups remarked how the messages made them less apprehensive about STI testing, particularly with the (new) knowledge that it didn't have to involve an invasive procedure.

... a lot of people when they like talk about STI testing, it's like, you know only at doctors, it's a big deal and you've got to feel embarrassed about doing it and the SMSs kind of put into context that it's not actually a stupid thing to do..... I haven't had one, but it made it seem a lot more easier to get one (Tracy, 17 years)

... I thought it was going to be the whole drop your daks* and everything, like yeah. If it's just a urine sample and a blood test then that's alright. (Bruce, 18 years) *pants

A few participants stated it made them consider getting an STI test, although they didn't necessarily go and get one.

Like think about it, like, "oh maybe I should get an STI test", eventually. (Brett, 17 years)

##### Consider Risk

A few participants felt that the information in some of the messages made them consider their risk of having an STI.

.. it makes you feel that you, that everybody could um, sort of have something in a way. Because usually you just assume that you don't have something because you, you feel okay or whatever. (Josh, 23 years)

#### Theme Three: Spreading the Word

Participants were asked if they shared the messages with others, whether by showing them the text or forwarding them on. Most had shared the messages, mainly with friends, family members and workmates. One participant did not show the messages to others, apart from a friend who also received the messages.

##### When and Why

Participants reported they were most likely to show other people the message if there were others around when they received the message, or if they found the message funny. A couple of females reported they also passed messages onto others when it was relevant to those individuals.

.... there was one that was really really funny and like I showed it to my friend and told him it was a joke but it was kind of like....it like jokingly like suited him in a way! [laughter] I told him like "someone sent me a message for you!" (Karen, 18 years)

In one focus group there was some discussion about it being easier to share messages that were linked into an event, because it gave a reason to pass the message on without risking accusing or offending individuals.

....it gives you a reason to pass it on because [it's] like an Easter greeting or a Valentines Day greeting (Mainisha, 22 years)

##### Reactions

Reactions from others were generally positive, although participants often needed to explain why they were receiving these messages.

They [people I work with] liked them. And they were like "how do you get these" and you have to like explain "I did this survey, and la la la" (Jess, 17 years)

Some participants commented they thought that reading out the messages to others help to reinforce the content within the message.

*Samuel (17 years): ..... you read them out to your friends and not only do you get the message across when you read out the message but then they ask you more about it*...

...

*Luke (18 years): And because, because you read it aloud you I think you remember it a lot more as well*.

## Discussion

Although a number of studies using SMS for health promotion have been published to date [6-16] this is the first evaluation to systematically examine how the characteristics of text messages influence the acceptability and efficacy of such interventions. As text messages continue to be used not only for health promotion, but also for other health-related functions such as disease self-management, appointment reminders, results of diagnostic testing and partner notification [3,20], it is critical to understand the factors that influence such intervention's success. Beyond SMS, understanding these factors is also relevant to other areas that could be exploited for health promotion purposes, such as updates on social networking sites, where concise formats are also required.

A clear finding from this evaluation was that the text messages were able to engage this group of young people for a subject area that is often viewed as 'sensitive' or 'personal'. The use of the SMS medium itself for health promotion was accepted, and was seen as being more personal than other approaches, particularly with the 'sign off' used.

However, this is a sophisticated and demanding audience- individuals wanted messages that were engaging, different, positive, contained a message relevant to them and were received at the right day and time. When these expectations were met, interest in the messages was high, and the messages were likely to be shared with others. If messages were deemed to be boring, too long, repetitive, use inappropriate language, or provide nothing new, they were ignored. Consistent with Giddens's 'reflexivity of knowledge',[1] participants were able to reflect on traditional health promotion strategies used to promote behaviour change, such as the use of fear, statistics and direct language and were able to see both the potential and drawbacks of these approaches. In a modern world where individuals are constantly exposed to a huge range of media, and are continually processing large amounts of information, sophisticated reflection on health messages presents a huge challenge for health promotion messages.

The quantitative findings from this study indicated a significant increase in knowledge after receiving the messages [17]; this finding was echoed in the focus groups with participants reporting obtaining new and specific information, as well as being reminded of information they already knew. The 'sign off' on the message gave the message credibility, increasing participant's trust of the information. In turn, this trust may increase the likelihood the information will be viewed favourably.

The reminding potential of SMS has been discussed previously where SMS has been used for appointment and medication reminders [3,20]. However this reminding function has also been seen as useful for health promotion related to smoking cessation [11,21] and may be more widely applicable to other behaviours. Reminders trigger individuals to think about potentially important topics that they might not otherwise consciously consider, or only consider at certain times, such as during a crisis. However the challenge is to find a way to provide a reminder that individuals want to receive, without being seen as repetitive, annoying or 'nagging' which will make them be less likely to a) pay attention to the message and b) continue to subscribe to receive the messages.

It appeared that participants did not believe the messages had any direct effects on their behaviour, but that the messages may have had some indirect effects. Most prominent was the changed perception of STI testing, with participants in almost all groups reporting they were now much less apprehensive about testing knowing that it was relatively easy and painless. This finding is consistent with previous work suggesting knowing STI testing is easy promotes screening uptake, while fear and anxiety about testing and testing being seen as difficult discourages uptake [22,23]. In addition, some participants reported the messages made them consider their risk of STIs, which is encouraging given young people are reported to underestimate their risk of infection [24,25]. The significant increase in STI testing observed in the quantitative evaluation of this project [17], as well as our previous randomised controlled trial [8], suggests that these perceived 'indirect' effects may have indeed had a direct effect, even without the messages providing direct referrals to clinical services.

Taken together, these findings suggest several elements need to be incorporated into short format health promotion messages to maximise the likelihood of behaviour change. All messages need to be novel or engaging (in both style or content) to ensure attention is given to them, and the individual needs to believe the sender of the message is a credible source of information. A balance between 'new' and 'reminding' content is required to ensure that content is delivered, but the messages are not viewed as repetitive and dismissed. The timing of message broadcast should be designed to be both acceptable to participants as well as relevant to the information provided within the messages.

This study has some limitations. Participants who were recruited to the focus groups had not unsubscribed from the messages, completed the online follow up survey and consented to be contacted again. Thus they may not be representative of all individuals who received the messages and there may be some bias in their responses, such as preferring our message style. Participants who preferred other styles of messages may have been less likely to be retained within the study and less likely to participate in the focus groups. The researchers conducting the focus groups and analysing the data were also involved in developing and delivering the intervention; this may have introduced some bias into the discussion or analysis. However participants were instructed at the outset of the discussion that we wished to receive all feedback (both positive and negative) and were repeatedly prompted to provide negative, as well as positive, feedback. Additionally, the group setting may have limited disclosure of any effects the messages may have had on behaviour change, as well as criticism of the messages (although negative feedback was actively encouraged).

## Conclusions

Mobile phones, in particular SMS, offer health promoters an exciting opportunity to engage personally with a huge number of individuals for low cost. This evaluation is the first to examine the characteristics of text messages that determine their acceptability and efficacy in promoting behaviour change. The key elements emerging, such as message style, language and broadcast schedule, are directly relevant to future studies using SMS for health promotion, as well as for future health promotion interventions in other 'new technology' mediums.

## Competing interests

The authors declare that they have no competing interests.

## Authors' contributions

JG contributed to the design of the focus groups, and was responsible for data collection, data analysis and manuscript preparation. ML contributed to the design of the focus groups, assisted with data collection and reviewed draft manuscripts. MH assisted with focus group design and provided comment on draft manuscripts. JH assisted with data collection and reviewed draft manuscripts. LK led the design of focus groups, oversaw data collection, data analysis and interpretation and assisted with manuscript preparation. All authors read and approved the final manuscript.

## Pre-publication history

The pre-publication history for this paper can be accessed here:

http://www.biomedcentral.com/1471-2458/10/792/prepub
